# Supplementary material for: Extracting entangled qubits from Majorana fermions in quantum dot chains through the measurement of parity
Source: Sci Rep. 2015 Jun 10;5:11188. doi: 10.1038/srep11188 (PMC5395956; doi:10.1038/srep11188)
Supplement: Supplementary Information [file srep11188-s1.pdf]

Supplementary materials for "Extracting entangled qubits from Majorana fermions in quantum dot chains through the measurement of parity"

Li Dai[1], Watson Kuo[1,2], and Ming-Chiang Chung[1,3,*]

[1]Department of Physics, National Chung Hsing University, Taichung, 40227, Taiwan
[2]Center of Nanoscience and Nanotechnology, and Institute of Nanoscience, National Chung Hsing University, Taichung 40227, Taiwan
[3]Physics Division, National Center for Theoretical Sciences, Hsinchu, 30013, Taiwan
[*]Correspondence to M.-C.C (mingchiangha@phys.nchu.edu.tw)

ABSTRACT

The supplementary materials consist of four sections. Sec. 1 discusses Bogoliubov-de Gennes transformation. Sec. 2 discusses the derivation of the ground states in real space of the chain. Sec. 3 gives an estimation of the electron-microwave coupling strength J. Sec. 4 discusses the teleportation for obtaining the final entangled qubits.

Bogoliubov-de Gennes transformation

One can use the Bogoliubov-de Gennes transformation[61] to solve for the zero mode

$$\tilde{b}_j = \frac{1}{2}\sum_{k=1}^{N}\left[\phi_{j,k}d_{2k-1} + i\psi_{j,k}d_{2k}\right] = \frac{1}{2}\sum_{k=1}^{N}\left[(\phi_{j,k}-\psi_{j,k})c_k^\dagger + (\phi_{j,k}+\psi_{j,k})c_k\right], \quad (j = 1,2,\cdots,N), \tag{1}$$

where $\phi_{j,k}$ and $\psi_{j,k}$ are real coefficients determined by the condition that the Hamiltonian (1) in the main text is diagonalized: $H = \sum_{j=1}^{N}\lambda_j(\tilde{b}_j^\dagger\tilde{b}_j - \frac{1}{2})$. We note that $[\tilde{b}_j, H] = \lambda_j\tilde{b}_j$. If there exists a zero mode, say $\lambda_N = 0$, we have $[\tilde{b}_N, H] = 0$. $[\tilde{b}_N, H]$ can be calculated by using Eq. (1) and (1). By requiring the coefficients of c_j and $c_j^\dagger$ in the calculated result to be zero, we get

$$\begin{pmatrix}\phi_{N,j+1}\\ \phi_{N,j}\end{pmatrix} = \begin{pmatrix}\frac{-\mu_j}{\Delta_j+w_j} & \frac{\Delta_{j-1}-w_{j-1}}{\Delta_j+w_j}\\ 1 & 0\end{pmatrix}\begin{pmatrix}\phi_{N,j}\\ \phi_{N,j-1}\end{pmatrix},$$
$$\begin{pmatrix}\psi_{N,j-1}\\ \psi_{N,j}\end{pmatrix} = \begin{pmatrix}\frac{-\mu_j}{\Delta_{j-1}+w_{j-1}} & \frac{\Delta_j-w_j}{\Delta_{j-1}+w_{j-1}}\\ 1 & 0\end{pmatrix}\begin{pmatrix}\psi_{N,j}\\ \psi_{N,j+1}\end{pmatrix},$$
$$(j = 1,2,\cdots,N), \tag{2}$$

where the variables with subscripts equal to $(N,0)$ or $(N,N+1)$ are assumed to be 0. For a uniform chain i.e. $w_j = w$, $\Delta_j = \Delta$ and $\mu_j = \mu$ in the above two equations, we notice that the transfer matrices are identical: $\begin{pmatrix}\frac{-\mu}{\Delta+w} & \frac{\Delta-w}{\Delta+w}\\ 1 & 0\end{pmatrix} \equiv A$. However, the index for $\phi_{N,i}$ will increase upon the action of the transfer matrix, while for $\psi_{N,i}$ the index will decrease. So, we can set $\psi_{N,j} = \phi_{N,N+1-j}$, and the Bogoliubov-de Gennes transformation (1) for $\tilde{b}_N$ becomes

$$\begin{aligned}\tilde{b}_N &= \frac{1}{2}\sum_{k=1}^{N}\left[\phi_{N,k}(d_{2k-1} + id_{2(N+1-k)})\right],\\ &= \frac{1}{2}\sum_{k=1}^{N}\left[\phi_{N,k}(c_k + c_k^\dagger + c_{N+1-k} - c_{N+1-k}^\dagger)\right]\end{aligned} \tag{3}$$

Suppose the two eigenvalues of the transfer matrix A are λ_1 and λ_2, i.e. $A|\lambda_i\rangle = \lambda_i|\lambda_i\rangle$, $i = 1,2$, so $A^{-1}A|\lambda_i\rangle = |\lambda_i\rangle = \lambda_i A^{-1}|\lambda_i\rangle$, thus the two eigenvalues of A^{-1} are λ_1^{-1} and λ_2^{-1} (for $\lambda_i \neq 0$). If $|\lambda_1| < 1$ and $|\lambda_2| < 1$ (or, $|\lambda_1^{-1}| < 1$ and $|\lambda_2^{-1}| < 1$), we will have

a decaying solution for $\phi_{N,i}$ (or $\phi_{N,N+1-i}$), $i = 1,2,\cdots,N$, in the thermodynamic limit $N \to \infty$. This corresponds to $|\mu| < 2w$ and $\Delta \neq 0$. For general values of w_j, Δ_j and μ_j, Ref.[36] proves that if w_j and Δ_j are sign-ordered i.e. sign($\Delta_j w_j$)=sign($\Delta_{j+1} w_{j+1}$), and $|\mu_j| < \max(|w_{j-1}|,|\Delta_{j-1}|)$, then the chain has zero-energy Majoarana fermions. The solution (4) can be verified by substituting it into Eq. (2). Actually, there is another solution: $\mu_1 = \mu_N = 0$, $\Delta_1 = -w_1$, $\Delta_{N-1} = -w_{N-1}$, corresponding to the absence of d_2 and d_{2N-2} in Eq. (3). The two MFs form a zero-energy edge mode that is similar to Eq. (5). Further analysis for this solution is also very similar to that for Eq. (4) and omitted.

Next, we prove the parity equality: $\prod_{j=1}^{N}(1-2c_j^\dagger c_j) = \prod_{j=1}^{N}(1-2\tilde{b}_j^\dagger \tilde{b}_j)$. The Hamiltonian (3) can be written as $H = \frac{i}{4}\sum_{l,m} A_{l,m} d_l d_m$. The coefficient matrix A is block diagonalized by a $2N \times 2N$ real orthogonal matrix W: $A_{j,k} = \sum_{m,n} W^T_{j,m}\Lambda_{m,n}W_{n,k}$, where Λ is a block diagonal matrix: $\Lambda_{2k-1,2k} = -\Lambda_{2k,2k-1} = \lambda_j$ (other matrix elements are zero). Note that $WW^T = W^TW = I$ so that $(\det W)^2 = \det W \det W^T = \det(WW^T) = 1$. When $\det W = 1$, the parity equality holds[38]. For the parameters in Eq. (4), we have the zero-energy mode in Eq. (5). The corresponding W will be $W_{j,1} = \delta_{j,2N-1}$, $W_{j,2N} = W_{2N,j} = \eta\delta_{j,2N}$, $W_{2N-1,j} = \delta_{j,1}$ for $1 \le j \le 2N$. While $W_{j,k} = (W_0)_{j,k-1}$ for $1 \le j \le 2N-2$, $2 \le k \le 2N-1$. Here $\eta = \det W_0$ is defined in Eq. (5). Therefore, $\det W$ can be calculated, according to the definition of determinant, as $\det W = \eta \det W_0 = \eta^2 = 1$. This concludes the proof.

Ground states in real space of the chain

In real space of the chain, $|G_1\rangle$ can be written as

$$|G_1\rangle = \sum_{i,j=0}^{1} z_{ij}|ij\rangle_{1,N}|\Psi_{ij}\rangle, \tag{4}$$

where we have grouped the states for the sites 1 and N together (assume $N \ge 3$ first), and $|\Psi_{ij}\rangle$ is the state for the sites from 2 to $N-1$. We notice that because $|G_1\rangle$ has an even parity (i.e. it is a superposition of the states with an even number of electrons), the states $|\Psi_{00}\rangle$ and $|\Psi_{11}\rangle$ also have an even parity, while the states $|\Psi_{01}\rangle$ and $|\Psi_{10}\rangle$ have an odd parity.

The state $|G_1\rangle$ satisfies $\tilde{b}_N|G_1\rangle = 0$. Using Eq. (5), we have $\tilde{b}_N|G_1\rangle$ equal to

$$\begin{aligned}&|00\rangle_{1,N}(z_{10}|\Psi_{10}\rangle - \eta z_{01}|\Psi_{01}\rangle) + |01\rangle_{1,N}(z_{11}|\Psi_{11}\rangle - \eta z_{00}|\Psi_{00}\rangle)\\ +&|10\rangle_{1,N}(z_{00}|\Psi_{00}\rangle - \eta z_{11}|\Psi_{11}\rangle) + |11\rangle_{1,N}(z_{01}|\Psi_{01}\rangle - \eta z_{10}|\Psi_{10}\rangle).\end{aligned}$$

We can set $z_{10} = \eta z_{01} \equiv x_1/\sqrt{2}$, $z_{00} = \eta z_{11} \equiv x_0/\sqrt{2}$, $|\Psi_{10}\rangle = |\Psi_{01}\rangle \equiv |S_e\rangle$, $|\Psi_{00}\rangle = |\Psi_{11}\rangle \equiv |S_o\rangle$ (note that $\eta^2 = 1$). Therefore, $|G_1\rangle$ equals

$$x_0\frac{|00\rangle_{1,N} + \eta|11\rangle_{1,N}}{\sqrt{2}}|S_e\rangle + x_1\frac{|10\rangle_{1,N} + \eta|01\rangle_{1,N}}{\sqrt{2}}|S_o\rangle, \tag{5}$$

We note that $\langle G_1|c_N^\dagger c_1^\dagger c_1 c_N|G_1\rangle = \frac{1}{2}|x_0|^2$. For the homogeneous chain ($\Delta_j = \Delta$, $w_j = w$, $\mu_j = \mu$), if we further have $\Delta = w > 0, \mu = 0$, the Hamiltonian (3) reduces to $H = iw\sum_{j=1}^{2N-1} d_{2j}d_{2j+1}$ and the new Dirac fermion operators that diagonalize the Hamiltonian (3) are $\tilde{b}_j = (c_{2j} + ic_{2j+1})/2$, $\tilde{b}_j^\dagger = (c_{2j} - ic_{2j+1})/2$, for $j = 1,2,\cdots,N-1$, ($\eta = 1$, see Ref.[38]). These relations together with Eq. (2) and (5) can be used to solve for c_1 and c_N. $c_1 = \frac{1}{2}(\tilde{b}_N + \tilde{b}_N^\dagger + i\tilde{b}_1 + i\tilde{b}_1^\dagger)$, $c_N = \frac{1}{2}(\tilde{b}_N - \tilde{b}_N^\dagger - i\tilde{b}_{N-1} + i\tilde{b}_{N-1}^\dagger)$. So $\langle G_1|c_N^\dagger c_1^\dagger c_1 c_N|G_1\rangle$ can be calculated by using these expressions and Eq. (6), and the result is $\frac{1}{4}$. Thus, x_0 can be chosen to be $\frac{\sqrt{2}}{2}$, and x_1 is determined by normalization condition of the state (8): $|x_0|^2 + |x_1|^2 = 1$, so $x_1 = \frac{\sqrt{2}}{2}$. For general values of Δ_j, w_j, μ_j, we only have Eq. (2) and (5), but $\tilde{b}_j$ ($j = 1,2,\cdots,N-1$) is unknown, which is a function of $c_k, c_k^\dagger$ ($k = 1,2,\cdots,N$) and can be obtained by diagonalizing the coefficient matrix in the Hamiltonian (1). Then, we can solve for c_1 and c_N to determine x_0, x_1, η in the state (8). The state $|G_2\rangle = \tilde{b}_N^\dagger|G_1\rangle$ by using Hermitian conjugate of Eq. (5). For the situation $N = 2$, the calculation is similar, where the two ground states are those before $|S_e\rangle$ and $|S_o\rangle$ in Eq. (5).

Estimation of J

The coupling between the microwave and the spin of the quantum dots in our proposal is analogous to the electron spin resonance which is usually weak as compared with the coupling through the electric dipole moment and thus ignored. However, in our experiment, this coupling is significant due to the presence of strong spin-orbit coupling in the quantum dots. We shall estimate the coupling strength J below. Note that the microwave is applied to the two boundary quantum dots which are decoupled from the inner part of the chain. Therefore, the superconducting proximity effect are not present in the measurement process.

The eigenstates and energy eigenvalues for the two spin-split levels of a single quantum dot are[36]

$$\begin{aligned} |\psi_\pm\rangle &= e^{-i(\frac{\pi x}{l_{so}}+\eta)\sigma_y}|\psi_0,\sigma_z=\pm 1\rangle, \\ E_\pm &= E_0-\frac{\pi^2}{2m^* l_{so}^2}\pm V_z\sqrt{c_0^2+s_0^2}, \end{aligned} \tag{6}$$

where $l_{so}=\frac{\pi}{m^*\alpha}$ (m^* the electron's effective mass, α the Rashba spin-orbit coupling strength), $\tan\eta = c_0/s_0-\sqrt{1+(c_0/s_0)^2}$ with $c_0=\langle\psi_0|\cos\frac{2\pi x}{l_{so}}|\psi_0\rangle$, $s_0=\langle\psi_0|\sin\frac{2\pi x}{l_{so}}|\psi_0\rangle$, E_0 and $|\psi_0\rangle$ are the eigen-energy and the spatial part of the eigen wave function without considering the magnetic field and the spin-orbit coupling, and V_z is the Zeeman potential.

With the measurement setup in Fig. 5, the Hamiltonian for a single quantum dot and the microwave changes to $\hbar\omega_m a^\dagger a+\frac{1}{2m^*}(\mathbf{p}+e\mathbf{A})^2-e\phi_m+\alpha\hat{\mathbf{z}}\cdot[(\mathbf{p}+e\mathbf{A})\times\sigma]+V(\mathbf{r})$, where $a^\dagger(a)$ is the creation (annihilation) operator of the microwave photons with frequency ω_m, $\mathbf{p}$ is the momentum of the electron, $\mathbf{A}$ and ϕ_m are the vector and scalar potentials of the microwave, $\sigma=(\sigma_x,\sigma_y,\sigma_z)$ is a vector of Pauli matrices, $V(\mathbf{r})$ is the confining potential. The term $-e\phi_m\sim e\mathbf{r}\cdot(\mathbf{E}+\partial_t\mathbf{A})$ describes the interaction of the electromagnetic filed with the dipole moment of the electron. This term is considered for coupled double quantum dots,[62] but it can be ignored here for a single quantum dot (or two decoupled quantum dots) because the only possible transition is that between the two energy levels in Eq. (6) which is electric-dipole forbidden ($\langle\psi_+|\mathbf{r}|\psi_-\rangle=0$). An exception is to use a third energy level to induce Raman transition,[63] but this method will not be considered here due to its incapability of realizing the dispersive coupling. One could also consider this third level and $|\psi_-\rangle$ forming a two-level system which allows transition through the electric dipole (usually with optical frequency). This method is feasible for achieving the dispersive coupling, but transitions involving other levels (e.g. from the aforementioned third level to $|\psi_+\rangle$) must be suppressed, otherwise spin-photon entanglement will be generated.[64] Instead, when restricted to the subspace spanned by $|\psi_\pm\rangle$, the relevant part describing the electron-microwave interaction in the Hamiltonian is $H_r=\frac{e}{m^*}(p_xA_x+p_yA_y+p_zA_z)+e\alpha(\sigma_yA_x-\sigma_xA_y)$. We assume that $\langle\psi_0|\mathbf{p}|\psi_0\rangle=0$, and notice that $\langle\psi_+|p_x|\psi_-\rangle=im^*\alpha$. The electromagnetic gauge is chosen to be[62,65] $\mathbf{A}=\frac{\mathbf{k}\times\mathbf{B_0}}{k^2}(a+a^\dagger)$, $\phi_m=\tilde{\phi}_m(a+a^\dagger)$, $\tilde{\phi}_m=\frac{l}{l_0}\sqrt{\frac{\hbar\omega_m}{c_{tot}}}$, where $\mathbf{k}$ is the wave vector of the microwave, $\mathbf{B_0}$ is the rms vacuum fluctuations of the magnetic field of the microwave, c_{tot} is the total capacitance of the transmission line resonator (TLR), a ($a^\dagger$) is the annihilation (creation) operator of the microwave photons, l is the coordinate along the electric field lines, and l_0 is the distance between the two planes facing the quantum dots (see Fig. 7). The second quantization of H_r with the rotating-wave approximation is $(f_j^\dagger c_j\langle\psi_+|H_r|\psi_-\rangle+h.c.)=Jf_j^\dagger c_j a+h.c.$.

$$J=e\alpha c_\eta|\mathbf{B_0}|/k, \tag{7}$$

where $c_\eta=-\langle\psi_0|\cos(\frac{2\pi x}{l_{so}}+2\eta)|\psi_0\rangle$. Experimentally, $\alpha=\pi/(m^*l_{so})$ with $m^*=0.023m_e$ for the InAs quantum dot, $l_{so}\sim 100$ nm, and $|\mathbf{B_0}|\sim|\nabla_l\tilde{\phi}_m|/c=\frac{1}{l_0c}\sqrt{\frac{\hbar\omega_m}{c_{tot}}}$ (c the speed of light), $\omega_m/2\pi\sim 10^{11}$ Hz, $c_{tot}\sim 1$ pF, $l_0\sim 5\ \mu$m, $k=2\pi/\lambda\sim 2\times 10^3$ m^{-1} and assume $\langle x|\psi_0\rangle=\sqrt{\frac{2}{L}}\sin(\frac{\pi x}{L})$ with $L\sim 120$ nm the width of the quantum dot in x direction, $J/2\pi$ can be tuned to 200 MHz. The phase ϕ in Eq. (11) can be generated through the time evolution $e^{it\delta f_j^\dagger f_j}f_j^\dagger e^{-it\delta f_j^\dagger f_j}=f_j^\dagger e^{it\delta}$ with $t=(\phi+2n\pi)/\delta$, n an integer. If we ignore the spin-orbit coupling, but instead consider the direct interaction of the microwave's magnetic field with the electron's spin via the Zeeman effect: $H_z=-\mu\cdot\mathbf{B}=\frac{e}{2m^*}\sigma\cdot\mathbf{B_0}(a+a^\dagger)$. The corresponding coupling strength after the second quantization and the rotating-wave approximation are performed will be $J_z=e|\mathbf{B_0}|/(2m^*)$. The ratio $J/J_z=2\alpha c_\eta m^*/k=c_\eta\lambda/l_{so}\sim 10^4$, indicating that the spin-microwave interaction will be considerably enhanced when the spin-orbit coupling is significant. We notice that this enhancement has already been pointed out in Ref.[47] of the main text, where the spin-photon coupling is realized in the interacting double quantum dots via the spin-orbit interaction. The coupling strength there ~ 0.4 MHz (see F.2. in the Supplemental Material of Ref.[47]) is much smaller than our value ~ 200 MHz. The main reason is that the spin rotation of the wave function in Ref.[47] is along the circumference of the carbon nanotube. For this configuration, the overlap between the microwave's vector potential and the electron's momentum vector with a varying direction (tangential to the circumference) is rather limited.

Teleportation

The state (9) will be useful for performing quantum teleportation if its parity is determined through the microwave measurement. Suppose the boundary sites are in the state: $\frac{1}{\sqrt{2}}(|00\rangle_{1,N}+|11\rangle_{1,N})$. We have an extra fermionic site with the index 0. The quantum state of this site may be correlated with its environment. Denote the state of the site 0 and its environment as $|\chi_0\rangle=y_0|0\rangle_0|\phi_0\rangle+y_1|1\rangle_0|\phi_1\rangle$, where $|0\rangle_0$ ($|1\rangle_0$) means that there is no (one) fermion in the site 0, y_0 and y_1 are the amplitudes, and $|\phi_0\rangle$ and $|\phi_1\rangle$ are some states of the environment. The task is to teleport the state in the site 0 to the site N. As we know, one need to perform Bell measurement. This seems not possible, but we can realize it indirectly through basis transformation in the Hilbert space of the site 0 and 1.

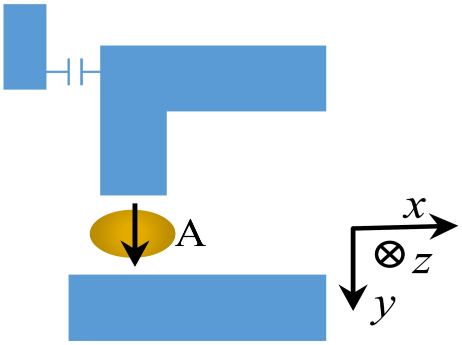

Figure 7. **The detailed diagram of the measuring setup.**

The initial state is

$$|\phi\rangle = |\chi_0\rangle \frac{|00\rangle_{1,N} + |11\rangle_{1,N}}{\sqrt{2}} \tag{8}$$

Then the interaction (hopping and pairing) between the sites 0 and 1 is switched on, and their chemical potential is finely tuned to be zero. The Hamiltonian is

$$H_{01} = -w_0(c_0^\dagger c_1 + c_1^\dagger c_0) + \Delta_0(c_0 c_1 + c_1^\dagger c_0^\dagger) \tag{9}$$

Choose the hopping amplitude w_0 and the superconducting gap Δ_0 to satisfy $w_0 = \Delta_0 > 0$. The time evolution operator is $\exp(-itH_{01})$. Setting $t = t_0 \equiv \frac{\pi}{4\Delta_0}$, we have

$$\begin{aligned} e^{-it_0 H_{01}}|\phi\rangle = \frac{1}{2}[&|00\rangle_{01}(y_0|0\rangle_N|\phi_0\rangle + iy_1|1\rangle_N|\phi_1\rangle) \\ &+|01\rangle_{01}(y_0|1\rangle_N|\phi_0\rangle + iy_1|0\rangle_N|\phi_1\rangle) \\ &+|10\rangle_{01}(iy_0|1\rangle_N|\phi_0\rangle + y_1|0\rangle_N|\phi_1\rangle) \\ &+|11\rangle_{01}(iy_0|0\rangle_N|\phi_0\rangle + y_1|1\rangle_N|\phi_1\rangle)], \end{aligned} \tag{10}$$

where we have written the states of the sites 0 and 1 first, and then the states of the site N and the environment of the site 0. It can be seen that we have four results when measuring the site 0 and 1 in the number basis: $|jk\rangle$, $j,k \in \{0,1\}$ (see Ref.[28] for the experimental realization of charge measurement). For each result, the corresponding state for the site N and the environment of the site 0 is equivalent to the original state $|\chi_0\rangle$ up to a local unitary transformation (gate) on the site N. The results 00 and 11 involve phase gates which are fulfilled by applying electric voltage, while the results 01 and 10 involve bit-flip gates which are realized by coupling the site N to a chain supporting zero-energy edge mode $\tilde{b}_m^\dagger \tilde{b}_m$. The chain has a Hamiltonian similar to Eq. (1) with parameters in Eq. (4), but N there is all replaced by another length m. The inner sites of a sub-chain in Fig. 1 can be chosen to serve as this chain. The coupling between the site N and the chain is[46]: $H_f = \kappa(c_N^\dagger - c_N)(\tilde{b}_m^\dagger + \tilde{b}_m)$, where κ denotes the coupling strength. We have $\exp(-itH_f)|0\rangle_N|G_1\rangle = -i|1\rangle_N|G_2\rangle$ and $\exp(-itH_f)|1\rangle_N|G_1\rangle = -i|0\rangle_N|G_2\rangle$ when $t = \pi/(2\kappa)$, realizing the bit-flip gate. Here $|G_1\rangle$ and $|G_2\rangle$ are the ground states of the chain (see Eq. (6) with N replaced by m), and their positions can be interchanged to obtain the other two Eqs. of time evolution.

For the other maximally entangled state $(|10\rangle_{1,N} + |01\rangle_{1,N})/\sqrt{2}$, the discussion is very similar. We only need to flip the state of the site N in Eq. (10) to obtain the result. To suppress the charge noise, it is preferred to use the basis of $|10\rangle$ and $|01\rangle$ rather than $|00\rangle$ and $|11\rangle$ of the double quantum dots for encoding the qubit. In this situation, the state $(|00\rangle_{1,N} + |11\rangle_{1,N})/\sqrt{2}$ and the pairing interaction in (9) should be avoided. When Δ_0 is set to be zero in (9), it can be verified that the teleportation succeeds with the probability $1/2$.

References

61. Lang, L.-J. & Chen, S. Majorana fermions in density-modulated p-wave superconducting wires. *Phys. Rev. B* **86**, 205135 (2012).

62. Childress, L., Sørensen, A. S. & Lukin, M. D. Mesoscopic cavity quantum electrodynamics with quantum dots. *Phys. Rev. A* **69**, 042302 (2004).

63. Imamoğlu, A. *et al.* Quantum information processing using quantum dot spins and cavity QED. *Phys. Rev. Lett.* **83**, 4204 (1999).

64. De Greve, K. *et al.* Quantum-dot spin-photon entanglement via frequency downconversion to telecom wavelength. *Nature* **491**, 421 (2012).

65. Kubo, Y. *et al.* Strong coupling of a spin ensemble to a superconducting resonator. *Phys. Rev. Lett.* **105**, 140502 (2010).
